# Quantitative assessment of temporal bone anatomy on preoperative CT in acquired cholesteatoma: validation of current methods and correlation to outcomes after surgery

**DOI:** 10.1007/s00405-026-10559-4

**Published:** 2026-08-26

**Authors:** L. Bengt van Rijssen, Maura C. Eggink, Jelle P. Algie, Maarten J. F. de Wolf, Erik van Spronsen

**Affiliations:** 1 Department of Otorhinolaryngology/Head and Neck Surgery, University Medical Center Groningen, University of Groningen, Groningen, The Netherlands; 2 Department of Otolaryngology/Head and Neck Surgery, Amsterdam UMC Location University of Amsterdam, Amsterdam, the Netherlands

**Keywords:** Cholesteatoma, Temporal bone anatomy, Validation, Computed tomography, Recidivism

## Abstract

**Objective:**

1. To identify methods of quantitative assessment of temporal bone anatomy on preoperative imaging in patients with primary acquired cholesteatoma.

2. To analyze the reproducibility of the identified methods.

3. To investigate whether quantitative assessment of temporal bone anatomy on preoperative imaging is correlated with recurrent or residual disease after cholesteatoma surgery.

**Methods:**

1. A literature review was performed.

2. External validation of the identified methods was achieved by investigating inter-rater reliability among three observers with varying otologic experience.

3. Methods were applied to CT scans of patients with primary acquired cholesteatoma and residual and/or recurrent disease (A), residual disease (B), or recurrent disease (C), and the results were compared with those of healthy controls (D).

**Results:**

Ten methods were identified and 23 patients in each of groups A, B and C and 25 patients in group D were included. Acceptable agreement between observers was reached for 7/10 methods. No significant differences were found by more than one observer between cases with residual or recurrent disease and cases without recidivism. Significant differences between cases with (A, B, and C) and without (D) cholesteatoma were found by all three observers for measurements of mastoid ventilation and lateral-to-medial height of the tegmen.

**Conclusions:**

We identified 10 measurements of anatomical dimensions of the temporal bone on preoperative imaging in patients with acquired cholesteatoma, and seven of these methods reached acceptable agreement between observers. Agreement was more common between the more experienced observers. None of the studied characteristics correlated with residual or recurrent cholesteatoma after surgery. Identifying preoperatively available risk factors for residual or recurrent cholesteatoma can aid patient counseling, surgical decision making and cholesteatoma staging systems.

**Supplementary Information:**

The online version contains supplementary material available at https://doi.org/10.1007/s00405-026-10559-4.

## Introduction

Cholesteatoma is the abnormal presence of skin and retention of keratin in the middle ear or temporal bone. Cholesteatoma growth may cause life threatening complications by infectious complications [1]. Treatment of cholesteatoma aims to eradicate disease and prevent residual or recurrent disease, with surgery being the treatment of choice in the vast majority of cases [2].

Various factors have been identified which influence rates of residual or recurrent disease (defined cumulatively as recidivism) after cholesteatoma surgery. Examples include patient-related factors such as age and disease related factors such as cholesteatoma localization and extension [3–9]. Varying dimensions of temporal bone anatomy have also been related to recidivism of cholesteatoma. For example, depth of the sinus tympani or mastoid pneumatization has previously been associated with residual disease [3, 9, 10].

Previous authors have made an effort to quantify or classify temporal bone anatomy on preoperative computed tomography imaging (CT). Examples include the size of the epitympanic recess, extension of the sinus tympani, configuration of the tegmen tympani and shape of the osseous external auditory canal [11–14]. Validation of the majority of these methods is lacking.

The goal of this study was threefold. First, to identify published measurements of anatomical dimensions of the temporal bone on preoperative imaging in patients with acquired cholesteatoma. Second, to validate these measurements by investigating inter-rater reliability and to determine if the results are reproduceable. Third, to investigate if these measurements are correlated to recurrent or residual disease after cholesteatoma surgery. This would be beneficial for preoperative patient counseling, surgical decision making and cholesteatoma staging systems.

## Materials and methods

### Literature review to identify relevant measurements

Firstly, pre-operative anatomical factors potentially influencing cholesteatoma surgery outcomes were identified in the literature [1–5]. A review of the literature was performed in PubMed Central in May 2024 using the following search entry: "CT"[tiab] AND ("cholesteatoma" OR "chronic otitis media" OR "CSOM" OR "chronic otitis externa") AND (("pneumatization"[tiab] AND ("temporal bone"[tiab] OR "mastoid"[tiab])) OR ("ventilation"[tiab] AND ("temporal bone"[tiab] OR "mastoid"[tiab])) OR "sinus tympani"[tiab] OR ("anterior epitympanic recess"[tiab] OR "anterior epitympanum"[tiab]) OR "tegmen"[tiab]). Cross-referencing was utilised to find additional articles. Inclusion criteria were: original English articles describing a quantitative method to asses or categorise temporal bone anatomy that could be predictive of recurrent or residual disease, using a relatively straightforward method on readily available sections. Methods describing anatomic changes as a result of cholesteatoma growth, such as ossicular erosion, were excluded. In case of multiple methods found per anatomical factor, the more precisely described method was chosen as this method was deemed more reproduceable. Studies using additional software to visualize or quantify anatomy were excluded, such as software for automated volume calculations. When the inclusion criteria were met based on the abstract, the full text was assessed. A similar article presenting a validated method to quantify the anatomy of the bony ear canal was included as a comparison.

### Participants and procedures

All patients who underwent surgery for primary acquired cholesteatoma between January 2009 and December 2022 at the Amsterdam University Medical Center, location Academic Medical Center in Amsterdam, the Netherlands, a tertiary otology referral center, were studied.

In total 4 groups were created. Group A consisted of cases without recidivism of cholesteatoma during follow up. Group B consisted of cases with residual disease. Residual disease results from incomplete surgical removal of the cholesteatoma matrix [15]. Residual cholesteatoma was found during routine second-look procedure or revision surgery indicated on a suspicious lesion on non-epi diffusion weighted magnetic resonance imaging. Group C consisted of cases with recurrent disease. Recurrent disease is the new formation of a retraction pocket after a complete previous surgical cholesteatoma removal [15]. Recurrent cholesteatoma was detected in the outpatient clinic or per-operatively during second-look surgery or ossicular chain reconstruction. In group A, B and C, patients were included with a minimum otoscopic and/or radiologic follow-up (FU) of 5 years. MRI-DWI scans were obtained as part of routine follow up approximately 1, 3 and 5 years post-operatively, or when indicated on the basis of clinical suspicion of disease.

Between groups A, B and C individual cases were matched based on age, affected side, and type of surgery as age and type of surgery are known confounders of cholesteatoma recidivism [6]. Control group D consisted of patients who received a cochlear implant between 2009 and 2022. Patients in the control group had no prior otologic surgery (including myringoplasty), no otologic complaints other than hearing loss and no congenital anomalies.

All patients in groups A, B and C underwent either canal wall up (CWU) procedure or canal wall up procedure with obliteration (CWUO). In the CWU procedure, a retro-auricular approach was performed, followed by a mastoidectomy ± scutum removal ± posterior tympanotomy (SAMEO classification S1A4M1a-1bExOx; S1A4M1a + 2aExOx; S1A4M1b + 2aExOx) [16]. In CWUO the mastoidectomy and epitympanic space were subsequently obliterated using various materials such as bone chips or hydroxy apatite (SAMEO classification S1A4M1a-1bExO2; S1A4M1a + 2aExO2; S1A4M1b + 2aExO2) [16].

### Measurements of anatomy

For each patient, various preoperative measurements of anatomy of the temporal bone were performed on computed tomography (CT) scans. CT scans were obtained as part of the routine work-up prior to surgery. Images were taken parallel to the orbitomeatal line. Slice thickness varied slightly between 0.4 mm and 0.6 mm.

Measurements were performed by 3 observers with varying clinical experience in otology: one otologist with > 15yrs of experience in academic otology (i.e. high experience), one senior resident in ENT-surgery specializing in otology (i.e. intermediate experience), and one junior resident in ENT-surgery (i.e. limited experience). Each observer had access to the methods as described in their original publications but received no additional explanation (e.g. from peers).

Measurements of preoperative anatomy were compared between the observers. Measurements with acceptable inter-observer agreement were compared between cases with (group B, C) and cases without residual or recurrent disease (group A). The measurements were also compared between cases with (group A, B and C) and without (group D) cholesteatoma to determine reproducibility of previous results.

### Statistical analysis

Continuous variables that followed normal and non-normal distributions were expressed as mean (standard deviation, SD) or median (interquartile range, IQR) values, respectively. Categorical variables were expressed as numbers (%). Baseline demographics were compared with an independent samples t-test for normally distributed data, a Chi-squared test for frequencies in one or more categories, and a Mann–Whitney U test for non-normally distributed data. Agreement between the three observers for categorical variables was evaluated using Fleiss κ. Agreement between two observers for categorical variables was evaluated using Cohen’s κ. Ninety-five percent confidence intervals (CI’s) are reported for κ. Agreement between the observers for continuous variables was evaluated using the intraclass correlation coefficient (ICC). Interpretation of Fleiss κ, Cohen’s κ and ICC was based on a classification provided by Landis and Koch: 0.0, poor; 0.0–0.20, slight; 0.21–0.40, fair; 0.41–0.60, moderate; 0.61–0.80, substantial; and 0.81–1.00, almost-perfect reproducibility [17]. At least substantial agreement (κ ≥ 0.6) was defined as acceptable [17–19]. Independent samples t-test was used to determine differences in continuous variables between cases in case of a normal distribution, and a Mann–Whitney-U test in case of a non-normal distribution. Statistical significance was set at *p* < 0.05. All analyses were performed using SPSS software (version 28.0; IBM Corp., Armonk, NY, USA).

## Results

### Identification of measurements

**Fig. 1:**
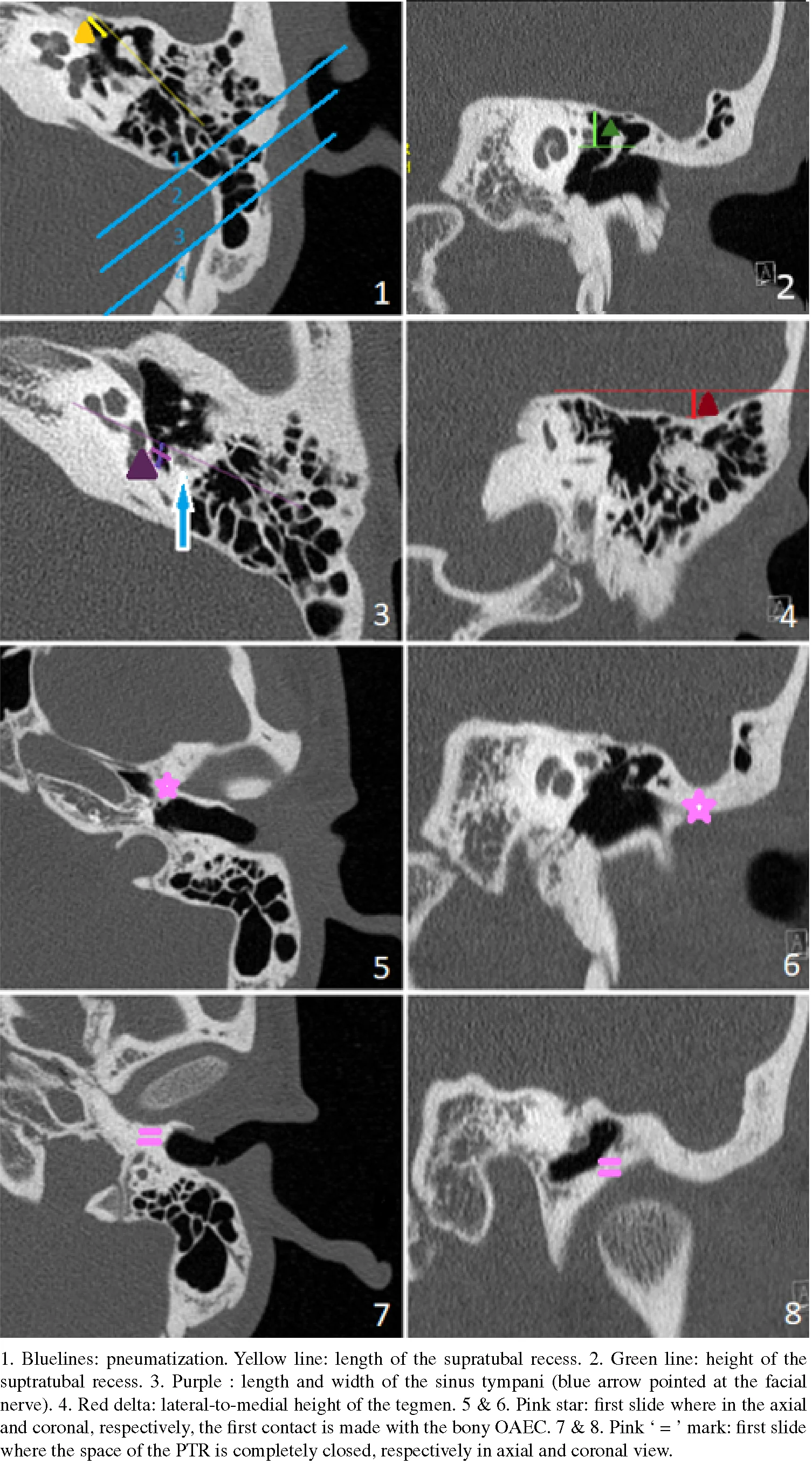
Preoperative measurements of temporal bone anatomy, demonstrated on a CT obtained in a healthy control patient (group D)

The literature review yielded 84 English articles. Seven articles matched the inclusion criteria, yielding ten measurements. The measurements are listed below and described in detail in Supplementary Table 1. In Fig. 1, the measurements are illustrated on CT slices of a healthy control from our study.

1. Mastoid pneumatization [20]
2. Mastoid ventilation [21]
3. Length of the supratubal recess (S1 length) [11]
4. Height of the supratubal recess (S1 height) [11]
5. Sinus tympani type (S2 type) [22]
6. Length of the sinus tympani (S2 length) [12]
7. Width of the sinus tympani (S2 width) [12]
8. Lateral-to-medial height of the tegmen (Tegmen LM post.) [13]
9. Depth of the pretympanic recess (depth of the PTR) [14]
10. Anterior curvature of the pretympanic recess (anterior curvature of the PTR) [14]

### Patient characteristics

In total 94 ears from 94 individual patients were included. This included 69 cholesteatoma cases after matching for age, affected side, and type of surgery: 23 (24,5%) cases without recidivism after surgery (group A), 23 cases with residual disease (group B) and 23 cases with recurrent disease (group C) after surgery. Twenty-five (26,6%, group D) control cases were included.

The range of ages included in each group (with average) was: group A 5–85 (34 average) years; group B 7–73 (28 average) years; group C 6–64 (33 average) years and group D 4–96 (31 average) years. These differences were not statistically significant (*p* > 0.05). Median (IQR) follow up in groups A, B and C was 9 (7–13) years. This was 8 (5–13) years in group A, 8 (6–12) years in group B, and 11 (8–13) years in group C.

### Validation of measurements

#### Inter-observer agreement

**Fig. 2:**
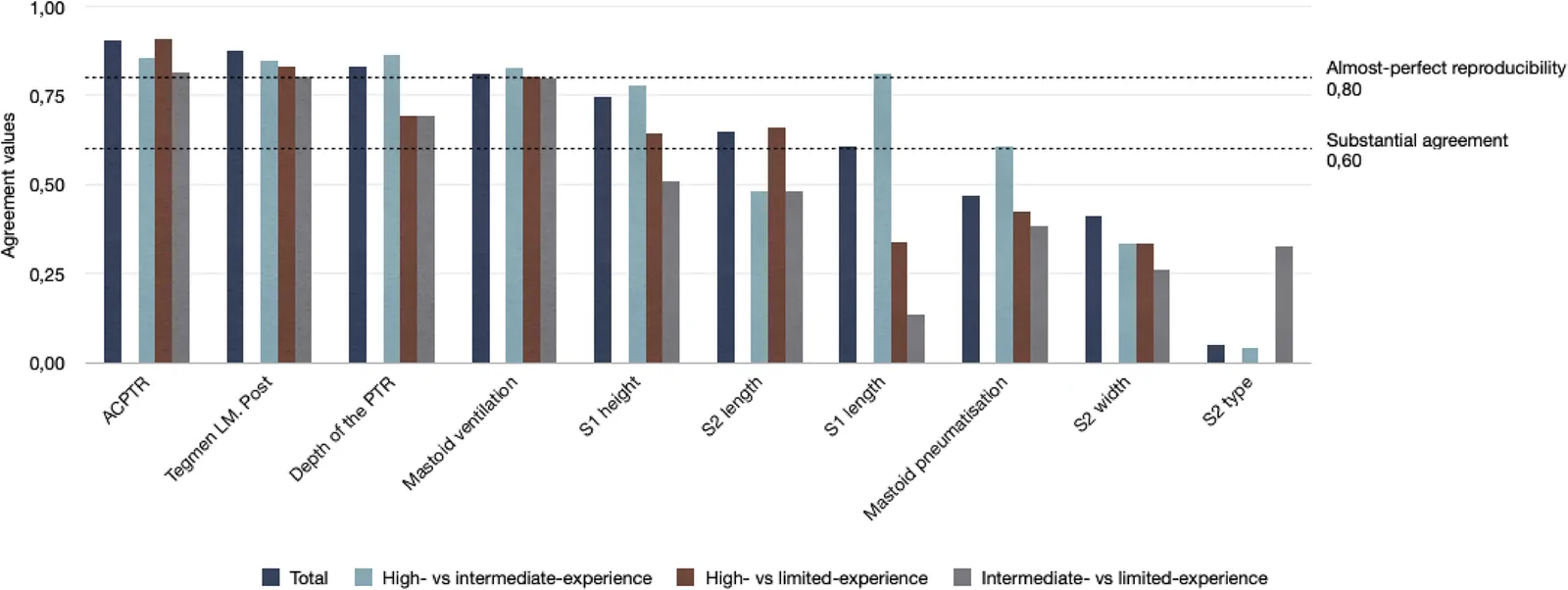
Interobserver agreement rates, in total and per experience level

Between the three observers, acceptable agreement was reached for 7/10 (70%) measurements (Fig. 2; Supplementary Table 2). Mastoid ventilation, lateral-to-medial height of the tegmen, depth of the PTR and anterior curvature of the PTR obtained almost-perfect reproducibility (Fleiss κ 0,81; ICC 0,88, 0,83 and 0,90, respectively). Substantial agreement was reached for S1 length, S1 height, and S2 length (ICC 0,61, 0,74 and 0,65, respectively). Acceptable agreement between observers was not reached for mastoid pneumatization, S2 type and S2 width and these measurements were therefore not used in further analysis (i.e. correlation of measurements with residual or recurrent cholesteatoma).

As is shown in Fig. 2, some measurements which obtained acceptable overall agreement rates between the three observers, reached different agreement levels between observers of varying experience. Between the high- and intermediate-experience observers acceptable agreement was reached for 7 (70%) measurements. Almost-perfect reproducibility was obtained for mastoid ventilation, S1 length, lateral-to-medial height of the tegmen, depth of the PTR and anterior curvature of the PTR (Cohen’s κ 0,83; ICC 0,81, 0,85, 0,86 and 0,86, respectively). Substantial agreement was obtained for mastoid pneumatization and S1 height (Cohen’s κ 0,61 and ICC 0,78, respectively). Between the high- and limited-experience observers acceptable agreement was reached for 6/10 (60%) measurements. Almost-perfect reproducibility was obtained for lateral-to-medial height of the tegmen and anterior curvature of the PTR (ICC 0,83 and 0,91). Substantial agreement was obtained for mastoid ventilation, S1 height, S2 length and depth of the PTR (Cohen’s κ 0,80; ICC 0,64, 0,66 and 0,69, respectively). Between the intermediate- and limited-experience observers acceptable agreement was reached for 4/10 (40%) measurements. Almost-perfect reproducibility was obtained for anterior curvature of the PTR (ICC 0,81). Substantial agreement was obtained for mastoid ventilation, lateral-to-medial height of the tegmen and depth of the PTR (Cohen’s κ 0,80 and ICC 0,80 and 0,69). Supplementary Table 3 demonstrates mean Cohen’s κ and ICC values with 95% CI for all measurements of agreement between the observers of varying experience level.

### Correlation of measurements to residual or recurrent cholesteatoma

#### Cases with residual or recurrent cholesteatoma versus no recidivism

**Fig. 3:**
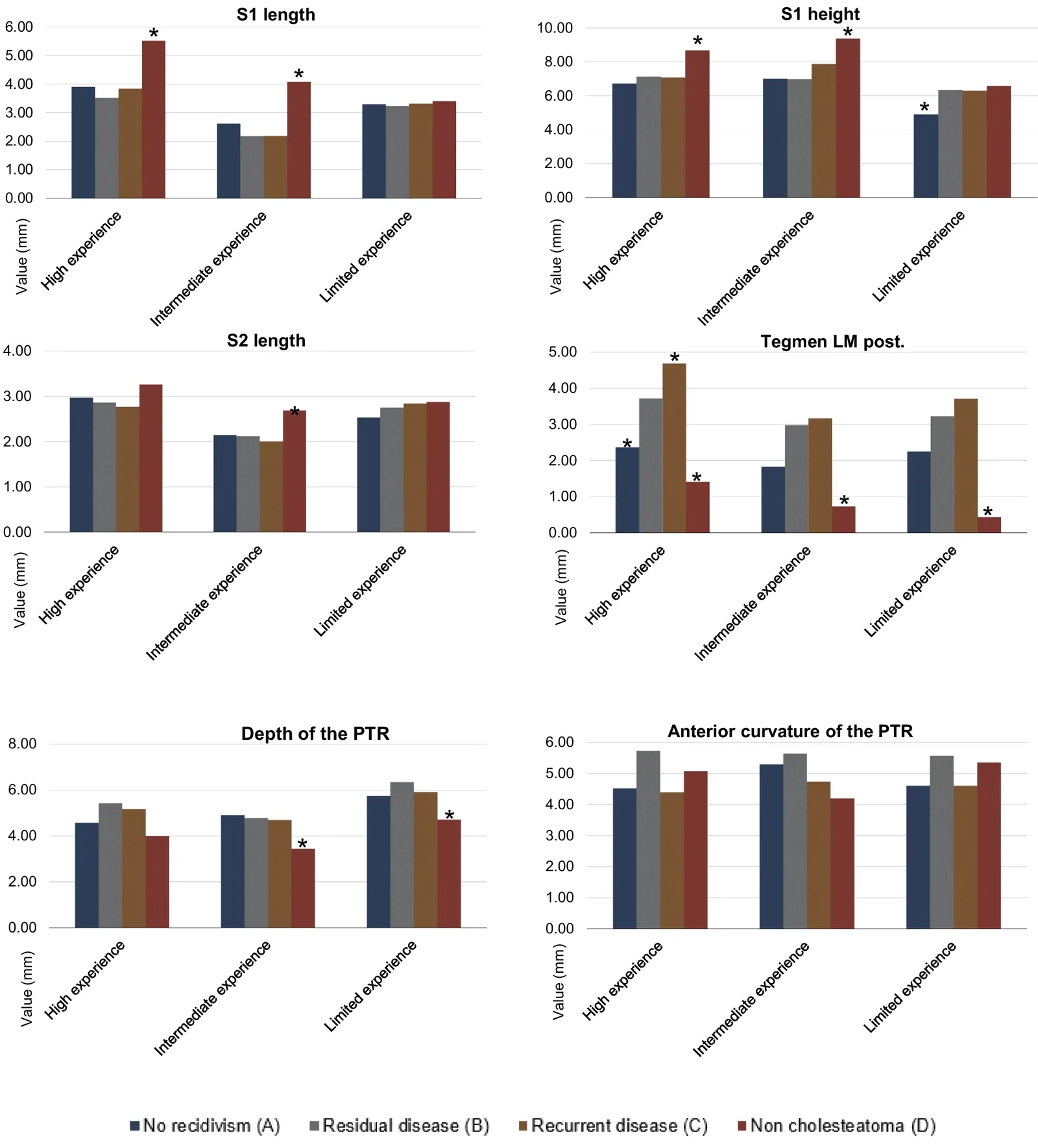

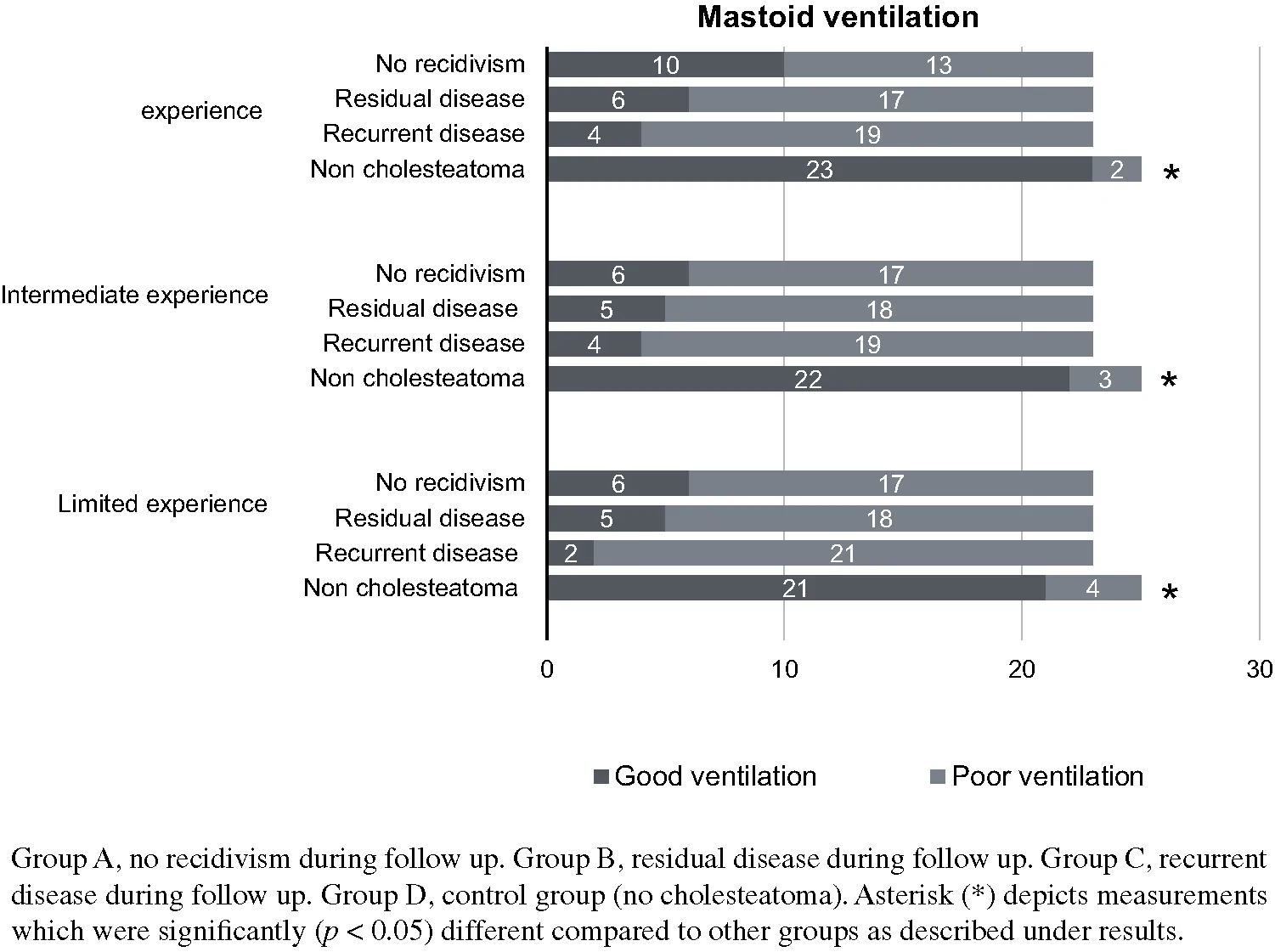
Differences in preoperative measurements of anatomy, stratified by observer experience. Only measurements which obtained acceptable agreement between the 3 observers are shown

As shown in Fig. 3 and Supplementary Table 5, no measurements were found with significant differences by all three, or two out of three observers when comparing cases with (group B + C) to cases without residual or recurrent disease (group A) after surgery. Significant differences were found by one observer for S1 height (limited-experience observer, *p* = 0,004) and lateral-to-medial height of the tegmen (high-experience observer, *p* = 0,01).

When comparing cases with residual disease (group B) to cases with no residual disease (i.e. without recidivism or with recurrent disease, group A + C) after surgery, no measurements were found with significant differences by any observer (Supplementary Table 6). When comparing cases with residual disease (group B) to cases without recidivism (i.e. group A), only a significant difference in the measurement of S1 height was found by one observer (limited-experience observer, *p* = 0,02).

No measurements were found with significant differences by all three, or two out of three observers when comparing cases with recurrent disease (group C) to cases with no recurrent disease (i.e. without recidivism or with residual disease, group A + B) after surgery (Supplementary Table 7). A significant difference in lateral-to-medial height of the tegmen was found by one observer (*p* = 0,02, high-experience observer). When comparing cases with recurrent disease (group C) to cases without recidivism (group A, i.e. not including cases with residual disease after surgery) significant differences in the measurements of S1 height, S2 length and lateral-to-medial height of the tegmen were found, each by one observer (S1 height; limited-experience observer, *p* = 0,004, S2 length; limited-experience observer, *p* = 0,04, lateral-to-medial height of the tegmen; high-experience observer, *p *= 0,004).

### Reproducibility of measurements

#### Cases with cholesteatoma versus healthy controls

Significant differences between cases with cholesteatoma (with or without recidivism after surgery, group A + B + C) and cases without cholesteatoma (control group D) were found by all three observers for measurements of mastoid ventilation (*p* < 0,001, *p* < 0,001 and *p* < 0,001) and lateral-to-medial height of the tegmen (*p* = 0,03, *p* = 0,01 and *p* = 0,01 for high-, intermediate-, and limited-experience observers, Supplementary Table 4). Significant differences were found by two out of three observers for S1 length, S1 height, and depth of the PTR (S1 length *p* < 0,001 for high- and intermediate-experience observers; S1 height *p* = 0,001 for high- and *p* < 0,001 for intermediate-experience observers; depth of the PTR *p* = 0,01 for intermediate-, *p* = 0,001 for limited-experience observers). A significant difference in S2 length was found by one observer (*p* = 0,03 for intermediate-experience observer).

## Discussion

Returning to the goals of this study, we identified 10 measurements of anatomical dimensions of the temporal bone on preoperative CT-imaging in patients with acquired cholesteatoma (goal 1). Acceptable inter-observer agreement rates were reached for 7 of the measurements: mastoid ventilation, lateral-to-medial height of the tegmen, depth of the pretympanic recess, anterior curvature of the pretympanic recess, anterior-to-posterior diameter of the supratubal recess, height of the supratubal recess, and length of the sinus tympani (goal 2). No significant differences in characteristics were found by more than one observer between cases with and cases without residual or recurrent cholesteatoma (goal 3).

### Validation of measurements

Quantitative assessment of temporal bone anatomy is not new; however, external validation by investigating inter-rater reliability of the above measurements was lacking. To our knowledge, only one of the methods was previously validated. Measurements of the osseous external ear canal (depth of the PTR and anterior curvature of the PTR) were previously demonstrated to have excellent agreement. [14]. One other study states very few discrepancies were found between observers in the measurement of mastoid pneumatisation [20]. Our study indicates the seven measurements of anatomical dimensions of temporal bone anatomy on preoperative CT-imaging which reached acceptable inter-observer agreement rates may be suitable for future clinical and research evaluation. The excellent agreement of measurements of the osseous external ear canal was confirmed in this study. Acceptable inter-observer agreement was not reached for 3 measurements. Different interpretations of the methods described in the original publications between the observers could play a role. Agreement was more often reached between the observers of high- and intermediate-experience. This suggests that experience affects the evaluation of temporal bone anatomy on CT-imaging. Therefore, when using these measurements in future research, experience should be taken into account.

### Correlation of measurements to residual or recurrent cholesteatoma

In this study the evaluated measurements were not able to predict the risk of cholesteatoma recidivism preoperatively. No significant differences in measurements were found by more than one observer between cases with and cases without residual or recurrent cholesteatoma.

Previous research has suggested a deep sinus tympani or a large anterior epitympanum to be related to residual disease [10, 11]. These areas were also defined as difficult access areas by the EAONO/JOS [15]. Our study did not find a relationship between the measurements of these difficult access areas on preoperative CT-imaging and residual disease. A possible explanation is that in the studied patients, the sinus tympani was routinely checked intraoperatively as a potential site of cholesteatoma location. Furthermore, in the majority of cases the incus and malleus head are removed to allow adequate visualization of the anterior epitympanic recess. Potentially, configuration rather than absolute size of these areas determines accessibility and subsequent complete eradication of disease. In the case of the sinus tympani, its position in regard to the facial nerve could be more determining in limiting exposure. Marchioni’s classification of the sinus tympani does take this into account and this classification was prognostic for residual disease, albeit in only the high-experience observer [22]. Furthermore, for this observer, type A sinus tympani surprisingly had a higher risk of residual disease than type B and C (supplementary Table 6). The classification also had a low agreement among observers in our study. A similar mechanism could be valid for the anterior epitympanum. As exposure of the anterior epitympanum is limited by the cog, the prominence of the cog might be a larger factor in residual disease than absolute size of the recess. Previously, a more prominent cog on CT was correlated to higher rates of cholesteatoma occurrence than in healthy controls, although this has not been related to specifically an anterior epitympanic localization of cholesteatoma or residual disease [23].

A significant difference in lateral-to-medial height of the tegmen was found by the high-experience observer in patients with recurrent disease compared to patients without recurrent disease, and in patients with recidivism compared to patients without recidivism. No correlation was found between a steeply sloping middle fossa dural plate and residual disease. We would expect a sloping middle fossa dural plate medially to hinder surgical exposure of the epitympanum and therefore potentially increase the occurrence of residual disease. The absolute distance between the middle fossa dural plate and the external ear canal was not taken in to account, but the measurement of lateral-to-medial height of the tegmen was previously found to be in good correlation with the distance from the tegmen to the external ear canal [13]. Recurrent disease was found to be more prevalent in patients with a larger lateral-to-medial height of the tegmen. The role of a low middle fossa dural plate in disease recurrence is subject to speculation, and may be the subject of further study.

Numerous factors related to recurrent or residual cholesteatoma have been studied. This has resulted in several staging systems which intend to demonstrate severity of disease and the difficulty to achieve complete surgical resection [15, 21, 24]. In previous research, however, these systems were found to have limited prognostic value [6, 9, 25, 26]. Furthermore, currently available staging systems usually depend on intra- and postoperative variables. This hampers utilization of the staging system for preoperative counselling of patients and surgical decision making. We therefore aimed to identify variables prognostic of recidivism available on preoperative CT. Unfortunately, this study did not identify factors to include in a future staging system.

### Correlation of measurements to the development of acquired cholesteatoma

Mastoid ventilation and lateral-to-medial height of the tegmen were identified to be prognostic of the development of an acquired cholesteatoma. Different dimensions of the supratubal recess, sinus tympani, and reduced mastoid pneumatization in patients with cholesteatoma have previously been shown to be associated with the development of an acquired cholesteatoma [11, 27]. Mastoid pneumatization was found to be prognostic for the development of an acquired cholesteatoma in our study by all 3 observers (Supplementary Table 4), but as no acceptable inter-rater reliability was reached for this measurement it was not included in our further analysis. In our study, length and height of the supratubal recess were also found to be associated with the development of an acquired cholesteatoma by the observers with high and intermediate experience. To our knowledge, no research exists examining the relationship between depth of the PTR and the occurrence of cholesteatoma. It could be hypothesized that a larger depth of the pre-tympanic recess hampers epithelial migration on the lateral tympanic membrane, potentially coupled with mucosal migration in the tympanic cavity or on the medial side of the tympanic membrane [28]. A role for the height of the tegmen in the pathogenesis of cholesteatoma is subject to speculation [29]. These may be subjects for further study.

### Strengths, weaknesses and future research

Strengths of our study include correction for known confounders such as age and type of surgery. Temporal bone pneumatization has been reported to progress rapidly until puberty followed by a slower, steady growth until early adulthood, while in some population groups mastoid air cell growth continues beyond early adulthood [30]. Age may therefore influence pneumatization, size of sinus tympani or anterior epitympanic recess, and configuration of the tegmen. By matching groups based on age, we mitigated the effect of this possible confounder as much as possible. However, age-related anatomical development remains a potential limitation and future studies may benefit from separate paediatric and adult analyses.

We did not investigate residual or recurrent cholesteatoma in specific anatomical sites but rather the occurrence of residual or recurrent disease in general. Keeping in mind the various theories on the development of cholesteatoma, each anatomical site could be hypothesized to influence recurrence [28]. Investigating the relationship between certain anatomic sites and residual disease at those sites would require large numbers of patients with residual disease at each site. There is interest in the occurrence of remote residuals, which makes it interesting to study residual disease for each location regardless of initial cholesteatoma location. Furthermore, several measurements such as mastoid ventilation and dimensions of the PTR are not related to primary cholesteatoma location.

Our study includes 23 patients in each cholesteatoma subgroup and 25 controls. The sample size may have limited the ability to detect smaller associations. Furthermore, false-negative findings cannot be excluded. The absence of significant associations should not be interpreted as definitive evidence that anatomical factors play no role in recidivism.

Agreement was more often reached between the observers of high- and intermediate-experience. This suggests that experience affects the evaluation of temporal bone anatomy on CT-imaging. We do not expect solely practicing otologists to use the studied measurements. With observers with varying levels of experience, the broad applicability of these methods was evaluated.

It is possible that the varying experience of observers, the number of cases included in each group and the limitations of the methods used to quantify temporal bone anatomy, influenced the outcomes of our study. It would therefore be valuable to repeat these quantifications in a larger dataset. This would allow for subgroup analyses of, for instance, paediatric patients, elucidating the role of age in temporal bone anatomy. Specific localisations of residual and recurrent disease could also be correlated to specific anatomic characteristics of the temporal bone. Once methods of quantification of temporal bone anatomy are found to be prognostic of residual or recurrent cholesteatoma, their measurements could be included in a future staging system.

## Conclusion

We identified 10 published measurements of anatomical dimensions of the temporal bone on preoperative imaging in patients with acquired cholesteatoma. Seven of these measurements demonstrated acceptable reproducibility and may be suitable for future clinical and research evaluation: mastoid ventilation, lateral-to-medial height of the tegmen, depth of the PTR, anterior curvature of the PTR, length of the supratubal recess, height of the supratubal recess, and length of the sinus tympani. Agreement was more common between the more experienced observers. None of the studied measurements were correlated to residual or recurrent cholesteatoma after surgery. It would be valuable to repeat these quantifications and expand on our findings in future studies as identifying risk factors for recidivism of cholesteatoma which are available preoperatively may aid surgical decision making, patient counseling, and future cholesteatoma staging systems.

## Supplementary Information

Below is the link to the electronic supplementary material.

Supplementary file1 (DOCX 25 KB)

Supplementary file2 (DOCX 16 KB)

Supplementary file3 (DOCX 18 KB)

Supplementary file4 (DOCX 22 KB)

Supplementary file5 (DOCX 23 KB)

Supplementary file6 (DOCX 23 KB)

Supplementary file7 (DOCX 23 KB)

## Data Availability

Datasets are not available.
