# Supplementary material for: Quantitative assessment of temporal bone anatomy on preoperative CT in acquired cholesteatoma: validation of current methods and correlation to outcomes after surgery
Source: Eur Arch Otorhinolaryngol. 2026 Aug 26;283(12):7791–800. doi: 10.1007/s00405-026-10559-4 (PMC13768221; doi:10.1007/s00405-026-10559-4)
Supplement: Supplementary file 1 [file 405_2026_10559_MOESM1_ESM.docx]

**Supplementary Table 1.** Identified measurements of anatomy in preoperative CT imaging of patients with primary acquired cholesteatoma. Copied largely from their original publications.

1. *Mastoid pneumatization (Han et al*.^18^)

“On the section where the malleoincudal complex appears as an ice cream-cone shape, three parallel lines angled at 45° in the anterolateral direction were applied in positions in which each line crossed the most anterior point of the sigmoid sinus at the junction with the petrous bone, the most lateral aspect along the transverse plane of the sigmoid groove, and the most posterior point of the sigmoid sinus, respectively.” Temporal bone pneumatization was then classified into four groups according to the degree of pneumatization in relation to these lines.

2. *Mastoid ventilation (adapted from Linder et al*^19^)

In the ChOLE classification, three classes of mastoid ventilation are described: more than 50% of cells aerated, more than 50% of cells opacified, or a sclerotic mastoid with no cells in the mastoid (except for possible attic ventilation). In this study, classification of mastoid ventilation was adapted from the ChOLE classification: more than 50% of cells aerated was defined as well ventilated, and more than 50% of cells opacified was defined as poorly ventilated.

3. *Length of the supratubal recess (S1 length, Marchioni et al.*^11^)

Measurements of the anterior-to-posterior diameter of the supratubal recess (S1 length) were performed on axial CT “parallel to the incudo-malleal axis for the deepest anterior-to-posterior (AP) diameter … selecting the most inferior slide that showed the Cog in its entirety”.

4. *Height of the supratubal recess (S1 height, Marchioni et al.*^11^)

Measurement of the height of the supratubal recess (S1 height) was measured on coronal CT as the distance “from the tegmen tympani to the cochleariform process”.

5. *Sinus tympani type (S2 type, Marchioni et al.*^20^)

Three types of sinus tympani were defined: type A (limited sinus tympani): “the medial boundary of the ST was lying in the same plane (parallel to the major axis of the temporal bone) with respect to the medial limit of the third portion of the facial nerve and so did not present a medial and posterior extension to the facial nerve”; type B (deep sinus tympani): “the medial boundary of the ST was lying medially with respect to the plane that runs parallel to the major axis of the temporal bone but not posteriorly with respect to the plane that runs through the medial limit of the third portion of the facial nerve” and type C (deep sinus tympani with posterior extension): “the medial boundary of the ST lies medially and posteriorly with respect to the third portion of the facial nerve”.

6. *Length of the sinus tympani (S2 length, El-Anwar et al.*^12^)

“The sinus tympani (ST) was identified in axial CT images with selection of the best image showing ST, promontory, and 3^rd^ portion of the facial nerve canal … The ST length (ST-L) was represented by the antero-posterior diameter of ST, from a tangential line passing through the anterior aspect of the facial nerve canal and the promontory to the deepest point of the ST”.

7. *Width of the sinus tympani (S2 width, El-Anwar et al.*^12^)

Measurement of sinus tympani was performed on the same axial CT images as for S2 length and S2 width was determined by the “widest transverse diameter of sinus tympani”.

8. *Lateral-to-medial height of the tegmen (Tegmen LM post., Makki et al.^13^)*

Measurement of the lateral-to-medial height of the tegmen was measured by using coronal CT “the slice in which the posterior limit of the superior semicircular canal first appears. The L-M post plane is defined from the beginning of the tegmen laterally to its level above the superior semicircular canal medially”. To determine lateral-to-medial height of the tegmen, a line was drawn at a tangent to the lowest point of the tegmen, and a line was also drawn at 90 degrees to this to the highest point on the tegmen.

Of note, some patients might display an upsloping, instead of a more common downsloping tegmen. Therefore, a negative value for this parameter was possible. A negative value for tegmen LM post was found in 9 patients by the high-experience observer, in 19 patients by the intermediate-experience observer, and 15 patients by the low-experience observer.

9. *Depth of the pretympanic recess (depth of the PTR, Van Spronsen et al.^14^)*

“Depth of the PTR (DPTR) was measured in an axial plane. First, the slide in which the inferior part of the OEAC [osseous external ear canal] was entirely visible was identified. Next, the number of slides inferiorly of this point was counted until the PTR was not visible anymore (i.e., a complete closure of the area in front of the tympanic membrane) … Then, the number of slides was multiplied by 0.55 mm (thickness of one slide), resulting in the DPTR.”

10. *Anterior curvature of the pretympanic recess (anterior curvature of the PTR, Van Spronsen et al.^14^)*

Measurement of the anterior curvature of the PTR was measured in the coronal plane. “Using the first slide in which the anterior part of the OEAC was completely visible, the starting point of the ACPTR was determined. Next, the number of slides was counted until the most anterior point next to the drum was reached … Finally, the number of slides was multiplied by 0.55 mm, resulting in the ACPTR”.
