# Supplementary material for: Quantitative assessment of temporal bone anatomy on preoperative CT in acquired cholesteatoma: validation of current methods and correlation to outcomes after surgery
Source: Eur Arch Otorhinolaryngol. 2026 Aug 26;283(12):7791–800. doi: 10.1007/s00405-026-10559-4 (PMC13768221; doi:10.1007/s00405-026-10559-4)
Supplement: Supplementary file 2 [file 405_2026_10559_MOESM2_ESM.docx]

**Supplementary table 2.** Interobserver agreement

| **Variable** | **Mean κ** | **Reproducibility** | **95% CI** |
| --- | --- | --- | --- |
| S2 type | 0,05 | Slight | -0,04 – 0,14 |
| Mastoid pneumatisation | 0,47 | Moderate | 0,40 – 0,54 |
| Mastoid ventilation | 0,81 | Almost-perfect | 0,69 – 0,93 |
|  |  |  |  |
|  | **Intraclass Correlation** |  |  |
| S1 length | 0,61 | Substantial | 0,45 – 0,73 |
| S1 height | 0,74 | Substantial | 0,64 – 0,82 |
| S2 length | 0,65 | Substantial | 0,50 – 0,75 |
| S2 width | 0,41 | Moderate | 0,17 – 0,59 |
| Tegmen LM post | 0,88 | Almost-perfect | 0,83 – 0,92 |
| Depth of the PTR | 0,83 | Almost-perfect | 0,76 – 0,88 |
| Anterior curvature of the PTR | 0,90 | Almost-perfect | 0,86 – 0,93 |

S2 type: sinus tympani type. S1 AP: anterior-to-posterior diameter of the supratubal recess. S1 SI: height of the supratubal recess. S2 length: length of the sinus tympani. S2 width: width of the sinus tympani. Tegmen LM post: lateral to medial height of the tegmen measured on the coronal CT slice in which the posterior limit of the superior semicircular canal first appears. DPTR: depth of the pretympanic recessus. ACPTR: anterior curvature of the pretympanic recessus.
