# Supplementary material for: Quantitative assessment of temporal bone anatomy on preoperative CT in acquired cholesteatoma: validation of current methods and correlation to outcomes after surgery
Source: Eur Arch Otorhinolaryngol. 2026 Aug 26;283(12):7791–800. doi: 10.1007/s00405-026-10559-4 (PMC13768221; doi:10.1007/s00405-026-10559-4)
Supplement: Supplementary file 3 [file 405_2026_10559_MOESM3_ESM.docx]

**Supplementary table 3.** Agreement between observers of varying experience level

| **Variable** | **High vs. Intermediate** | **Reprod.** |  | **High vs limited** | **Reprod.** |  | **Intermediate vs limited** | **Reprod.** |
| --- | --- | --- | --- | --- | --- | --- | --- | --- |
|  | *Mean κ* |  |  | *Mean κ* |  |  | *Mean κ* |  |
| S2 type | 0,04 | Slight |  | 0,000 | Poor |  | 0,33 | Fair |
| Mastoid pneum. | 0,61 | Substantial |  | 0,42 | Moderate |  | 0,39 | Fair |
| Mastoid vent. | 0,83 | Almost-perfect |  | 0,80 | Almost-perfect |  | 0,80 | Substantial |
|  |  |  |  |  |  |  |  |  |
|  | *Intraclass Correlation (ICC)* |  |  | *ICC* |  |  | *ICC* |  |
| S1 length | 0,81 | Almost-perfect |  | 0,34 | Fair |  | 0,14 | Slight |
| S1 height | 0,78 | Substantial |  | 0,64 | Substantial |  | 0,51 | Moderate |
| S2 length | 0,48 | Moderate |  | 0,66 | Substantial |  | 0,48 | Moderate |
| S2 width | 0,34 | Fair |  | 0,34 | Fair |  | 0,26 | Fair |
| Tegmen LM. post | 0,85 | Almost-perfect |  | 0,83 | Almost-perfect |  | 0,80 | Almost-perfect |
| Depth of the PTR | 0,86 | Almost-perfect |  | 0,69 | Substantial |  | 0,69 | Substantial |
| Anterior curvature PTR | 0,86 | Almost-perfect |  | 0,91 | Almost-perfect |  | 0,81 | Almost-perfect |

S2 type: sinus tympani type. S1 AP: anterior-to-posterior diameter of the supratubal recess. S1 SI: height of the supratubal recess. S2 length: length of the sinus tympani. S2 width: width of the sinus tympani. Tegmen LM post: lateral to medial height of the tegmen measured on the coronal CT slice in which the posterior limit of the superior semicircular canal first appears. DPTR: depth of the pretympanic recessus. ACPTR: anterior curvature of the pretympanic recessus.
