# Supplementary material for: Quantitative assessment of temporal bone anatomy on preoperative CT in acquired cholesteatoma: validation of current methods and correlation to outcomes after surgery
Source: Eur Arch Otorhinolaryngol. 2026 Aug 26;283(12):7791–800. doi: 10.1007/s00405-026-10559-4 (PMC13768221; doi:10.1007/s00405-026-10559-4)
Supplement: Supplementary file 4 [file 405_2026_10559_MOESM4_ESM.docx]

**Supplementary table 4.** Measurements of anatomy in cases without (n=25) compared to cases with cholesteatoma (n=69) by observers of varying experience level.

a) High experience

|  | **Cholesteatoma** | |  |
| --- | --- | --- | --- |
| **Variable** | *No (nr)*  *Group D* | *Yes (nr)*  *Group A, B, C* | *P-value* |
| S2 type  A  B  C | 3  11  11 | 12  39  18 | 0,25 |
| Mastoid pneumatisation  Hypo  Moderate  Good  Hyper | 1  3  4  17 | 18  17  18  16 | <0,001 |
| Mastoid ventilation  Good  Poor | 23  2 | 20  49 | <0,001 |
|  |  |  |  |
|  | *No (mm)* | *Yes (mm)* | *P-value* |
| S1 length | 5,52 | 3,76 | <0,001 |
| S1 height | 8,68 | 6,99 | 0,001 |
| S2 length | 3,26 | 2,87 | 0,12 |
| S2 width | 2,84 | 3,13 | 0,25 |
| Tegmen LM post | 1,41 | 3,60 | 0,03 |
| Depth of the PTR | 4,00 | 5,10 | 0,051 |
| Anterior curvature of the PTR | 5,08 | 4,88 | 0,81 |

b) Intermediate experience

|  | **Cholesteatoma** | |  |
| --- | --- | --- | --- |
| **Variable** | *No (nr)*  *Group D* | *Yes (nr)*  *Group A, B, C* | *P-value* |
| S2 type  A  B  C | 11  13  1 | 53  13  3 | 0,006 |
| Mastoid pneumatisation  Hypo  Moderate  Good  Hyper | 1  1  4  19 | 18  16  17  18 | <0,001 |
| Mastoid ventilation  Good  Poor | 22  3 | 15  54 | <0,001 |
|  |  |  |  |
|  | *No (mm)* | *Yes (mm)* | *P-value* |
| S1 length | 4,08 | 2,32 | <0,001 |
| S1 height | 9,38 | 7,30 | <0,001 |
| S2 length | 2,68 | 2,09 | 0,03 |
| S2 width | 1,90 | 1,78 | 0,50 |
| Tegmen LM post | -0,73 | 2,66 | 0,01 |
| Depth of the PTR | 3,44 | 4,80 | 0,01 |
| Anterior curvature of the PTR | 4,20 | 5,23 | 0,10 |

c) Limited experience

|  | **Cholesteatoma** | |  |
| --- | --- | --- | --- |
| **Variable** | *No (nr)*  *Group D* | *Yes (nr)*  *Group A, B, C* | *P-value* |
| S2 type  A  B  C | 9  14  2 | 37  30  2 | 0,24 |
| Mastoid pneumatisation  Hypo  Moderate  Good  Hyper | 1  3  3  18 | 5  21  17  26 | 0,03 |
| Mastoid ventilation  Good  Poor | 21  4 | 13  56 | <0,001 |
|  |  |  |  |
|  | *No (mm)* | *Yes (mm)* | *P-value* |
| S1 length | 3,40 | 3,28 | 0,66 |
| S1 height | 6,59 | 5,84 | 0,11 |
| S2 length | 2,87 | 2,71 | 0,47 |
| S2 width | 1,74 | 1,96 | 0,19 |
| Tegmen LM post | 0,43 | 3,06 | 0,01 |
| Depth of the PTR | 4,72 | 6,00 | 0,001 |
| Anterior curvature of the PTR | 5,36 | 4,93 | 0,50 |

S2 type: sinus tympani type. S1 AP: anterior-to-posterior diameter of the supratubal recess. S1 SI: height of the supratubal recess. S2 length: length of the sinus tympani. S2 width: width of the sinus tympani. Tegmen LM post: lateral to medial height of the tegmen measured on the coronal CT slice in which the posterior limit of the superior semicircular canal first appears. DPTR: depth of the pretympanic recessus. ACPTR: anterior curvature of the pretympanic recessus.
