# Supplementary material for: Quantitative assessment of temporal bone anatomy on preoperative CT in acquired cholesteatoma: validation of current methods and correlation to outcomes after surgery
Source: Eur Arch Otorhinolaryngol. 2026 Aug 26;283(12):7791–800. doi: 10.1007/s00405-026-10559-4 (PMC13768221; doi:10.1007/s00405-026-10559-4)
Supplement: Supplementary file 5 [file 405_2026_10559_MOESM5_ESM.docx]

**Supplementary table 5.** Ears without (n=23) compared to ears with (n=46) recidivism of cholesteatoma by observers of varying experience level.

a) High experience

|  | **Recidivism** | |  |
| --- | --- | --- | --- |
| **Variable** | *No (nr)*  *Group A* | *Yes (nr)*  *Group B, C* | *P-value* |
| S2 type  A  B  C | 3  16  4 | 9  23  14 | 0,30 |
| Mastoid pneumatisation  Hypo  Moderate  Good  Hyper | 7  5  7  4 | 11  12  11  12 | 0,77 |
| Mastoid ventilation  Good  Poor | 10  13 | 10  36 | 0,06 |
|  |  |  |  |
|  | *No (mm)* | *Yes (mm)* | *P-value* |
| S1 length | 3,90 | 3,68 | 0,47 |
| S1 height | 6,73 | 7,11 | 0,47 |
| S2 length | 2,97 | 2,82 | 0,61 |
| S2 width | 3,07 | 3,16 | 0,75 |
| Tegmen LM post | 2,37 | 4,20 | 0,02 |
| Depth of the PTR | 4,57 | 5,30 | 0,30 |
| Anterior curvature of the PTR | 4,52 | 5,07 | 0,50 |

b) Intermediate experience

|  | **Recidivism** | |  |
| --- | --- | --- | --- |
| **Variable** | *No (nr)*  *Group A* | *Yes (nr)*  *Group B, C* | *P-value* |
| S2 type  A  B  C | 15  6  2 | 38  7  1 | 0,22 |
| Mastoid pneumatisation  Hypo  Moderate  Good  Hyper | 4  6  8  5 | 14  10  9  13 | 0,42 |
| Mastoid ventilation  Good  Poor | 6  17 | 9  37 | 0,54 |
|  |  |  |  |
|  | *No (mm)* | *Yes (mm)* | *P-value* |
| S1 length | 2,61 | 2,18 | 0,14 |
| S1 height | 7,01 | 7,43 | 0,48 |
| S2 length | 2,14 | 2,06 | 0,64 |
| S2 width | 1,69 | 1,82 | 0,51 |
| Tegmen LM post | 1,83 | 3,07 | 0,18 |
| Depth of the PTR | 4,91 | 4,74 | 0,81 |
| Anterior curvature of the PTR | 5,30 | 5,19 | 0,88 |

c) Limited experience

|  | **Recidivism** | |  |
| --- | --- | --- | --- |
| **Variable** | *No (nr)*  *Group A* | *Yes (nr)*  *Group B, C* | *P-value* |
| S2 type  A  B  C | 12  9  2 | 25  21  0 | 0,13 |
| Mastoid pneumatisation  Hypo  Moderate  Good  Hyper | 2  7  7  7 | 3  14  10  19 | 0,79 |
| Mastoid ventilation  Good  Poor | 6  17 | 7  39 | 0,28 |
|  |  |  |  |
|  | *No (mm)* | *Yes (mm)* | *P-value* |
| S1 length | 3,30 | 3,28 | 0,95 |
| S1 height | 4,89 | 6,32 | <0,001 |
| S2 length | 2,53 | 2,78 | 0,11 |
| S2 width | 2,08 | 1,90 | 0,46 |
| Tegmen LM post | 2,25 | 3,47 | 0,17 |
| Depth of the PTR | 5,74 | 6,13 | 0,45 |
| Anterior curvature of the PTR | 4,61 | 5,09 | 0,48 |

S2 type: sinus tympani type. S1 AP: anterior-to-posterior diameter of the supratubal recess. S1 SI: height of the supratubal recess. S2 length: length of the sinus tympani. S2 width: width of the sinus tympani. Tegmen LM post: lateral to medial height of the tegmen measured on the coronal CT slice in which the posterior limit of the superior semicircular canal first appears. DPTR: depth of the pretympanic recessus. ACPTR: anterior curvature of the pretympanic recessus.
