# Supplementary material for: Quantitative assessment of temporal bone anatomy on preoperative CT in acquired cholesteatoma: validation of current methods and correlation to outcomes after surgery
Source: Eur Arch Otorhinolaryngol. 2026 Aug 26;283(12):7791–800. doi: 10.1007/s00405-026-10559-4 (PMC13768221; doi:10.1007/s00405-026-10559-4)
Supplement: Supplementary file 6 [file 405_2026_10559_MOESM6_ESM.docx]

**Supplementary table 6.** Ears with residual disease (n=23) compared to no residual disease (i.e. without recidivism or with recurrent disease after surgery, n=46) by observers of varying experience level.

a) high experience

|  | **Residual disease** | |  |
| --- | --- | --- | --- |
| **Variable** | *No (nr)*  *Group A, C* | *Yes (nr)*  *Group B* | *P-value* |
| S2 type  A  B  C | 5  31  10 | 7  8  8 | 0,03 |
| Mastoid pneumatisation  Hypo  Moderate  Good  Hyper | 13  11  14  8 | 5  6  4  8 | 0,35 |
| Mastoid ventilation  Good  Poor | 14  32 | 6  17 | 0,71 |
|  |  |  |  |
|  | *No (mm)* | *Yes (mm)* | *P-value* |
| S1 length | 3,88 | 3,52 | 0,27 |
| S1 height | 6,91 | 7,14 | 0,73 |
| S2 length | 2,87 | 2,86 | 0,98 |
| S2 width | 3,17 | 3,04 | 0,70 |
| Tegmen LM post | 3,53 | 3,71 | 0,80 |
| Depth of the PTR | 4,87 | 5,43 | 0,48 |
| Anterior curvature of the PTR | 4,46 | 5,74 | 0,20 |

b) Intermediate experience

|  | **Residual disease** | |  |
| --- | --- | --- | --- |
| **Variable** | *No (nr)*  *Group A, C* | *Yes (nr)*  *Group B* | *P-value* |
| S2 type  A  B  C | 35  8  3 | 18  5  0 | 0,44 |
| Mastoid pneumatisation  Hypo  Moderate  Good  Hyper | 10  12  14  10 | 8  4  3  8 | 0,22 |
| Mastoid ventilation  Good  Poor | 10  36 | 5  18 | 1,00 |
|  |  |  |  |
|  | *No (mm)* | *Yes (mm)* | *P-value* |
| S1 length | 2,40 | 2,17 | 0,47 |
| S1 height | 7,45 | 6,98 | 0,45 |
| S2 length | 2,07 | 2,12 | 0,80 |
| S2 width | 1,82 | 1,69 | 0,53 |
| Tegmen LM post | 2,50 | 2,98 | 0,61 |
| Depth of the PTR | 4,80 | 4,78 | 0,97 |
| Anterior curvature of the PTR | 5,02 | 5,64 | 0,51 |

c) Limited experience

|  | **Residual disease** | |  |
| --- | --- | --- | --- |
| **Variable** | *No (nr)*  *Group A, C* | *Yes (nr)*  *Group B* | *P-value* |
| S2 type  A  B  C | 27  17  2 | 10  13  0 | 0,22 |
| Mastoid pneumatisation  Hypo  Moderate  Good  Hyper | 4  14  12  16 | 1  7  5  10 | 0,84 |
| Mastoid ventilation  Good  Poor | 8  38 | 5  18 | 0,66 |
|  |  |  |  |
|  | *No (mm)* | *Yes (mm)* | *P-value* |
| S1 length | 3,31 | 3,24 | 0,82 |
| S1 height | 5,60 | 6,34 | 0,20 |
| S2 length | 2,69 | 2,75 | 0,73 |
| S2 width | 1,94 | 2,00 | 0,83 |
| Tegmen LM post | 2,98 | 3,23 | 0,77 |
| Depth of the PTR | 5,83 | 6,35 | 0,38 |
| Anterior curvature of the PTR | 4,61 | 5,57 | 0,25 |

S2 type: sinus tympani type. S1 AP: anterior-to-posterior diameter of the supratubal recess. S1 SI: height of the supratubal recess. S2 length: length of the sinus tympani. S2 width: width of the sinus tympani. Tegmen LM post: lateral to medial height of the tegmen measured on the coronal CT slice in which the posterior limit of the superior semicircular canal first appears. DPTR: depth of the pretympanic recessus. ACPTR: anterior curvature of the pretympanic recessus.
