# Supplementary material for: Quantitative assessment of temporal bone anatomy on preoperative CT in acquired cholesteatoma: validation of current methods and correlation to outcomes after surgery
Source: Eur Arch Otorhinolaryngol. 2026 Aug 26;283(12):7791–800. doi: 10.1007/s00405-026-10559-4 (PMC13768221; doi:10.1007/s00405-026-10559-4)
Supplement: Supplementary file 7 [file 405_2026_10559_MOESM7_ESM.docx]

**Supplementary table 7.** Ears with recurrent disease (n=23) compared to no recurrent disease (i.e. without recidivism or with residual disease after surgery, n=46) by observers of varying experience level.

a) High experience

|  | **Recurrent disease** | |  |
| --- | --- | --- | --- |
| **Variable** | *No (nr)*  *Group A, B* | *Yes (nr)*  *Group C* | *P-value* |
| S2 type  A  B  C | 10  24  12 | 2  15  6 | 0,38 |
| Mastoid pneumatisation  Hypo  Moderate  Good  Hyper | 12  11  11  12 | 6  6  7  4 | 0,85 |
| Mastoid ventilation  Good  Poor | 16  30 | 4  19 | 0,13 |
|  |  |  |  |
|  | *No (mm)* | *Yes (mm)* | *P-value* |
| S1 length | 3,71 | 3,85 | 0,59 |
| S1 height | 6,94 | 7,09 | 0,77 |
| S2 length | 2,92 | 2,77 | 0,58 |
| S2 width | 3,05 | 3,27 | 0,48 |
| Tegmen LM post | 3,04 | 4,69 | 0,01 |
| Depth of the PTR | 5,00 | 5,17 | 0,80 |
| Anterior curvature of the PTR | 5,13 | 4,39 | 0,34 |

b) Intermediate experience

|  | **Recurrent disease** | |  |
| --- | --- | --- | --- |
| **Variable** | *No (nr)*  *Group A, B* | *Yes (nr)*  *Group C* | *P-value* |
| S2 type  A  B  C | 33  11  2 | 20  2  1 | 0,31 |
| Mastoid pneumatisation  Hypo  Moderate  Good  Hyper | 12  10  11  13 | 6  6  6  5 | 0,85 |
| Mastoid ventilation  Good  Poor | 11  35 | 4  19 | 0,54 |
|  |  |  |  |
|  | *No (mm)* | *Yes (mm)* | *P-value* |
| S1 length | 2,39 | 2,18 | 0,36 |
| S1 height | 7,00 | 7,88 | 0,16 |
| S2 length | 2,13 | 2,00 | 0,47 |
| S2 width | 1,69 | 1,95 | 0,25 |
| Tegmen LM post | 2,40 | 3,17 | 0,44 |
| Depth of the PTR | 4,85 | 4,70 | 0,79 |
| Anterior curvature of the PTR | 5,47 | 4,74 | 0,44 |

c) Limited experience

|  | **Recurrent disease** | |  |
| --- | --- | --- | --- |
| **Variable** | *No (nr)*  *Group A, B* | *Yes (nr)*  *Group C* | *P-value* |
| S2 type  A  B  C | 22  22  2 | 15  8  0 | 0,29 |
| Mastoid pneumatisation  Hypo  Moderate  Good  Hyper | 3  14  12  17 | 2  7  5  9 | 0,97 |
| Mastoid ventilation  Good  Poor | 11  35 | 2  21 | 0,13 |
|  |  |  |  |
|  | *No (mm)* | *Yes (mm)* | *P-value* |
| S1 length | 3,27 | 3,32 | 0,89 |
| S1 height | 5,61 | 6,30 | 0,16 |
| S2 length | 2,64 | 2,84 | 0,20 |
| S2 width | 2,04 | 1,80 | 0,18 |
| Tegmen LM post | 2,74 | 3,71 | 0,25 |
| Depth of the PTR | 6,04 | 5,91 | 0,79 |
| Anterior curvature of the PTR | 5,09 | 4,61 | 0,43 |

S2 type: sinus tympani type. S1 AP: anterior-to-posterior diameter of the supratubal recess. S1 SI: height of the supratubal recess. S2 length: length of the sinus tympani. S2 width: width of the sinus tympani. Tegmen LM post: lateral to medial height of the tegmen measured on the coronal CT slice in which the posterior limit of the superior semicircular canal first appears. DPTR: depth of the pretympanic recessus. ACPTR: anterior curvature of the pretympanic recessus.
